# Prognostic impact of pan-immune inflammation value in small-cell lung cancer treated with chemoradiotherapy and prophylactic cranial irradiation

**DOI:** 10.17305/bb.2025.12669

**Published:** 2025-07-03

**Authors:** Aybala Nur Ucgul, Huseyin Hazir, Huseyin Bora

**Affiliations:** 1 Department of Radiation Oncology, Gulhane Training and Research Hospital, Ankara, Türkiye; 2 Department of Radiation Oncology, Faculty of Medicine, Gazi University, Ankara, Türkiye

**Keywords:** Small-cell lung cancer, SCLC, pan-immune inflammation value, PIV, chemoradiotherapy, CRT, prophylactic cranial irradiation, PCI, prognosis

## Abstract

Determining prognosis is crucial for treatment selection, especially for prophylactic cranial irradiation (PCI), in patients with limited-stage small-cell lung cancer (LS-SCLC). This study evaluates the prognostic value of the pan-immune inflammation value (PIV) in patients with LS-SCLC. We included patients who underwent thoracic chemoradiotherapy (TRT) and PCI at our clinic between July 2012 and April 2024. PIV was calculated as (neutrophil count × platelet count × monocyte count)/lymphocyte count. Receiver operating characteristic (ROC) curve analysis was used to determine the optimal pre-treatment PIV cut-off to divide patients into two groups. Survival outcomes between these groups were compared using Kaplan–Meier analysis and log-rank tests. Multivariate analyses were conducted using Cox regression. Fifty-nine patients were included in the study. The optimal PIV cut-off was identified as 911 (AUC: 0.60, Sensitivity: 0.31, Specificity: 0.94, J-index: 0.26). Patients were grouped based on PIV levels: low (<911) and high (≥911). Lower PIV levels were significantly associated with improved overall survival (OS) (39 months vs 10 months, *P* < 0.001) and intracranial progression-free survival (ICPFS) (not reached vs 15 months, *P* < 0.001). The independent prognostic value of PIV was confirmed in multivariate analyses for both OS (*P* < 0.001) and ICPFS (*P* < 0.001). These findings suggest that pre-treatment PIV is an independent prognostic marker in LS-SCLC patients undergoing TRT and PCI.

## Introduction

Small-cell lung cancer (SCLC) accounts for 15% of all lung cancers and is associated with a poor prognosis due to its rapid growth and early spread [1, 2]. In addition to the American Joint Committee on Cancer (AJCC) TNM staging system, the Veterans Administration Lung Study Group (VALSG) classification system categorizes SCLC into two stages: limited stage and extensive stage [3, 4]. For limited-stage SCLC (LS-SCLC), the standard treatment is thoracic chemoradiotherapy (CRT), while chemotherapy is the standard treatment for extensive-stage SCLC (ES-SCLC) [5, 6]. However, the two-year overall survival (OS) rate is 40% for LS-SCLC, compared to less than 10% for ES-SCLC [7]. The use of prophylactic cranial irradiation (PCI) remains controversial in patients who respond to initial treatment in the magnetic resonance imaging (MRI) and chemotherapy-immunotherapy era [8]. Therefore, identifying prognostic markers is crucial for selecting effective treatment modalities.

Following the identification of the impact of systemic inflammation on tumors, several inflammation indices have been developed as prognostic markers for various cancers [4, 9]. Among these, the most frequently used markers are the neutrophil/lymphocyte ratio (NLR), platelet/lymphocyte ratio (PLR), and lymphocyte/monocyte ratio (LMR) [10]. However, these indices only account for two parameters. As a result, newer indices have been developed that incorporate additional parameters, such as the systemic immune inflammation index (SII) and the pan-immune inflammation value (PIV) [11, 12]. Fucà et al. developed the PIV as a new prognostic biomarker for patients with metastatic colorectal cancer. PIV is calculated using the formula: (neutrophil count × platelet count × monocyte count)/lymphocyte count. Their findings indicated that patients with a high PIV had poorer survival outcomes [12]. Since then, several studies have evaluated the use of PIV in various cancers, including colorectal [13], esophageal [14, 15], and breast cancer [16, 17].

Although many studies have explored the prognostic significance of PIV in non-SCLC (NSCLC), research on its role in SCLC remains limited [4, 18, 19]. The objective of this retrospective study was to evaluate the prognostic significance of PIV in patients with LS-SCLC who have undergone thoracic CRT and PCI.

## Materials and methods

### Patients

A retrospective analysis was conducted on a cohort of patients with LS-SCLC who underwent thoracic CRT and PCI at our clinic between July 2012 and April 2024. The study population was identified from our institutional records. This study included patients who met the following criteria: age between 18 and 85 years, an Eastern Cooperative Oncology Group (ECOG) performance status of 0–2, and a pathological diagnosis of SCLC. Staging was categorized as limited-stage according to the VALSG classification. Patients with immune system disorders or a history of immunosuppressive medication were excluded from the study.

### Treatment protocols

The standard treatment for SCLC at our clinic consists of thoracic radiotherapy with a total dose of 60 Gy (2 Gy per fraction, over 30 days) and 4–6 cycles of cisplatin-etoposide chemotherapy (cisplatin 60 mg/m^2^ IV on day 1 and etoposide 120 mg/m^2^ IV on days 1–3, administered every 28 days). For patients who cannot tolerate cisplatin, carboplatin and etoposide are used instead. A total of 43 patients received cisplatin-etoposide, while 16 received carboplatin-etoposide. TRT generally begins concurrently with the first cycles of chemotherapy.

Patients who respond to thoracic CRT and have no metastases on brain MRI receive PCI with a total dose of 25 Gy (2.5 Gy per fraction, over 10 days). Responses were categorized as either complete response (CR) or partial response (PR). A CR was defined as the disappearance of all target lesions with no new lesions observed, while a PR was defined as a ≥30% decrease in the sum of diameters of target lesions.

### PIV measurements

Pre-treatment PIV was calculated from blood samples collected within one week prior to the initiation of thoracic CRT, using the formula: (neutrophil count × platelet count × monocyte count)/lymphocyte count.

### Follow-up

In accordance with institutional protocols, patients were examined using brain MRI and thoracic CT scans at three-month intervals for the first two years, followed by scans every six months for the subsequent 2–5 years, or earlier if new symptoms developed.

### Outcomes

The primary objective of this study was to evaluate the relationship between PIV and OS, defined as the time from the start of CRT until death or the last visit. The secondary objective was to assess the association between PIV and various survival metrics: local recurrence-free survival (LRFS) and intracranial progression-free survival (ICPFS). LRFS is defined as the duration from the start of CRT until local progression, death, or the last visit, while ICPFS is defined as the time until brain metastasis, death, or the last visit.

### Ethical statement

This investigation was approved by the Ethics Committee of Gulhane Training and Research Hospital (Approval No: 2025/128, Date: 12 June 2025). This study was conducted in accordance with the Declaration of Helsinki.

### Statistical analysis

Analysis was conducted using IBM SPSS 27. To determine the optimal cut-off value for pre-treatment PIV, receiver operating characteristic (ROC) analysis was performed. Patients were categorized into two groups: Low PIV and High PIV. The characteristics of all patients in the cohort, as well as those in the two groups, were assessed using independent samples *t*-tests and chi-squared tests. Survival analyses were performed using Kaplan–Meier survival curves and log-rank tests. For multivariate analysis, Cox regression was performed. All tests were conducted with a 95% confidence interval, and a *P* value of less than 0.05 was considered statistically significant.

## Results

The patient and treatment characteristics are summarized in Table 1. The median age of the patients was 59 years, with the majority being male. Most patients had an ECOG performance score of 0–1. In line with the clinical protocol, most patients received concurrent CRT.

**Table 1:** Patient characteristics based on PIV levels

	**Whole Cohort**	**Low PIV (<911)**	**High PIV (≥911)**	***P* value**
Median age, years (range)	59 (46–79)	59.5 (46–79)	58 (56–68)	0.92
*Age, n (%)*				
<59 years	30 (50.8)	25 (50)	5 (55.5)	0.75
≥59 years	29 (49.2)	25 (50)	4 (44.5)	
*Gender, n (%)*				
Male	47 (79.7)	39 (78)	8 (88.9)	0.45
Female	12 (20.3)	11 (22)	1 (11.1)	
*ECOG performance score, n (%)*				
0	9 (15.2)	5 (10)	4 (44.5)	**0.02**
1	47 (79.7)	42 (84)	5 (55.5)	
2	3 (5.1)	3 (6)	0 (0)	
*Comorbidity, n (%)*				
Yes	29 (49.2)	25 (50)	4 (44.5)	0.75
No	30 (50.8)	25 (50)	5 (55.5)	
*Smoking, n (%)*				
Yes	37 (62.7)	31 (62)	6 (66.7)	0.79
No	22 (37.3)	19 (38)	3 (33.3)	
*Smoking packs/year, n (%)*				
<30 packs/year	12 (32.4)	9 (29)	3 (50)	0.31
≥30 packs/year	25 (67.6)	22 (71)	3 (50)	
*Cht Regimen, n (%)*	0.64			
Cisplatin-Etoposide	43 (72.9)	37 (74)	6 (66.7)	
Carboplatin-Etoposide	16 (27.1)	13 (26)	3 (33.3)	
*Number of Cht Cycles, n (%)*				
<6 cycles	28 (47.5)	26 (52)	2 (22.2)	0.10
≥6 cycles	31 (52.5)	24 (48)	7 (77.8)	
*CRT, n (%)*	0.98			
Concurrent	46 (78)	39 (78)	7 (77.8)	
Sequential	13 (22)	11 (22)	2 (22.2)	

ECOG: Eastern Cooperative Oncology Group; Cht: Chemotherapy; CRT: Chemoradiotherapy; PIV: Pan-immune inflammation value.

The optimal cut-off value for pre-treatment PIV was determined to be 911 using ROC analysis (AUC: 0.60, Sensitivity: 0.31, Specificity: 0.94, J-index: 0.26). Patients were divided into two groups based on their pre-treatment PIV: those with PIV < 911 (low PIV group) and those with PIV ≥ 911 (high PIV group). No statistically significant differences were observed between the two groups regarding patient and treatment characteristics, except for the ECOG performance score.

Among the 59 patients included in the study, 18 (30.5%) achieved a CR, and 41 (69.5%) achieved a PR on thoracic CT following CRT. Notably, all CRs were observed in the low PIV group, while no patients in the high PIV group achieved CR (*P* ═ 0.031).

**Figure 1.**
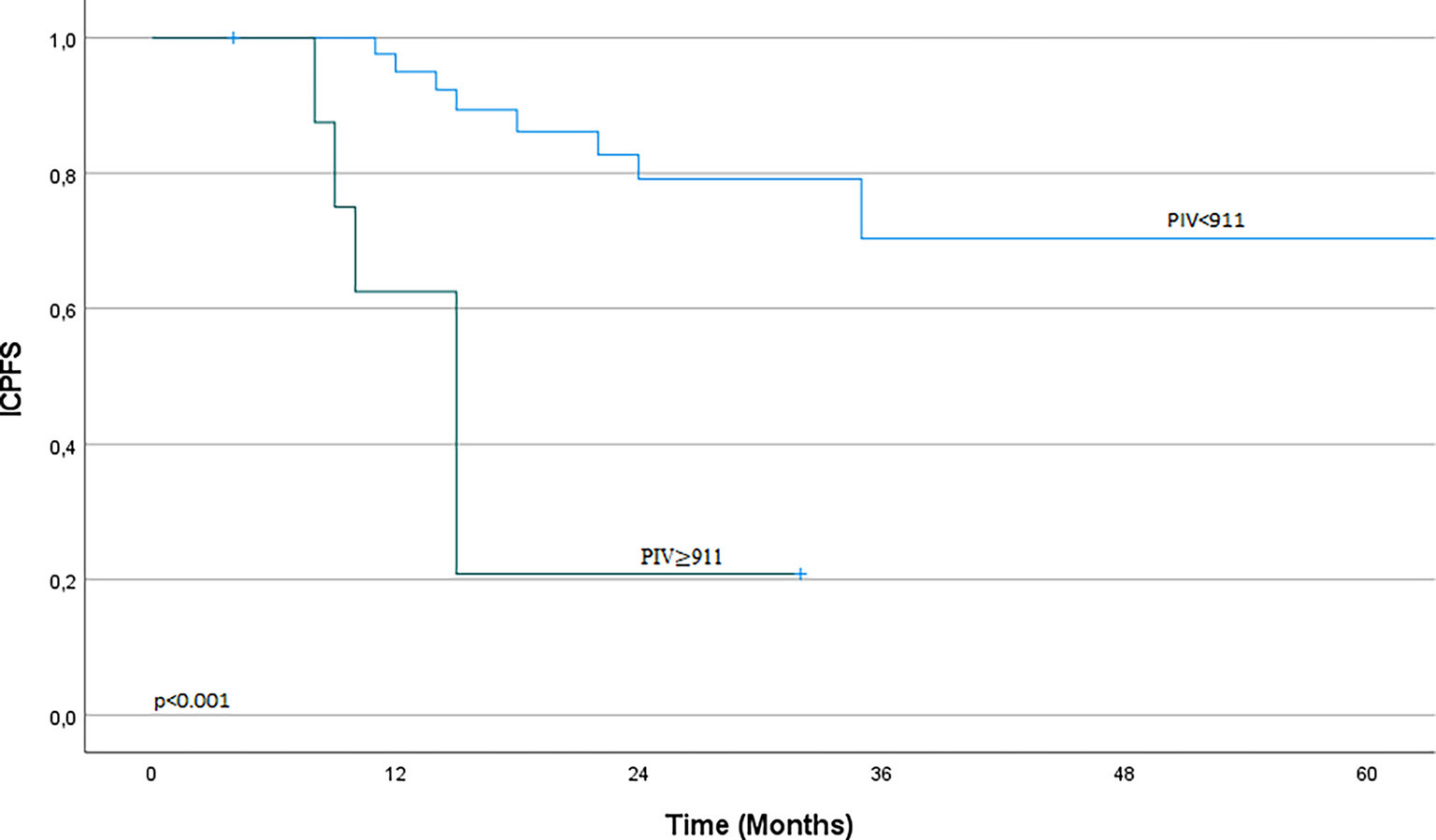
**The relationship between pre-treatment PIV and ICPFS.** Kaplan–Meier survival curve showing ICPFS stratified by PIV groups. Patients with PIV < 911 demonstrated significantly longer ICPFS compared to those with PIV ≥ 911 (*P* < 0.001). The median ICPFS was not reached in the low PIV group, while it was 15 months in the high PIV group. ICPFS: Intracranial progression-free survival; PIV: Pan-Immune inflammation value.

No statistically significant differences in LRFS were found between the low and high PIV groups (*P* ═ 0.30). However, ICPFS was significantly higher in the low PIV group compared to the high PIV group. The median ICPFS was not reached for the low PIV group, while it was 15 months for the high PIV group (*P* < 0.001). Figure 1 illustrates the ICPFS survival curve.

**Figure 2.**
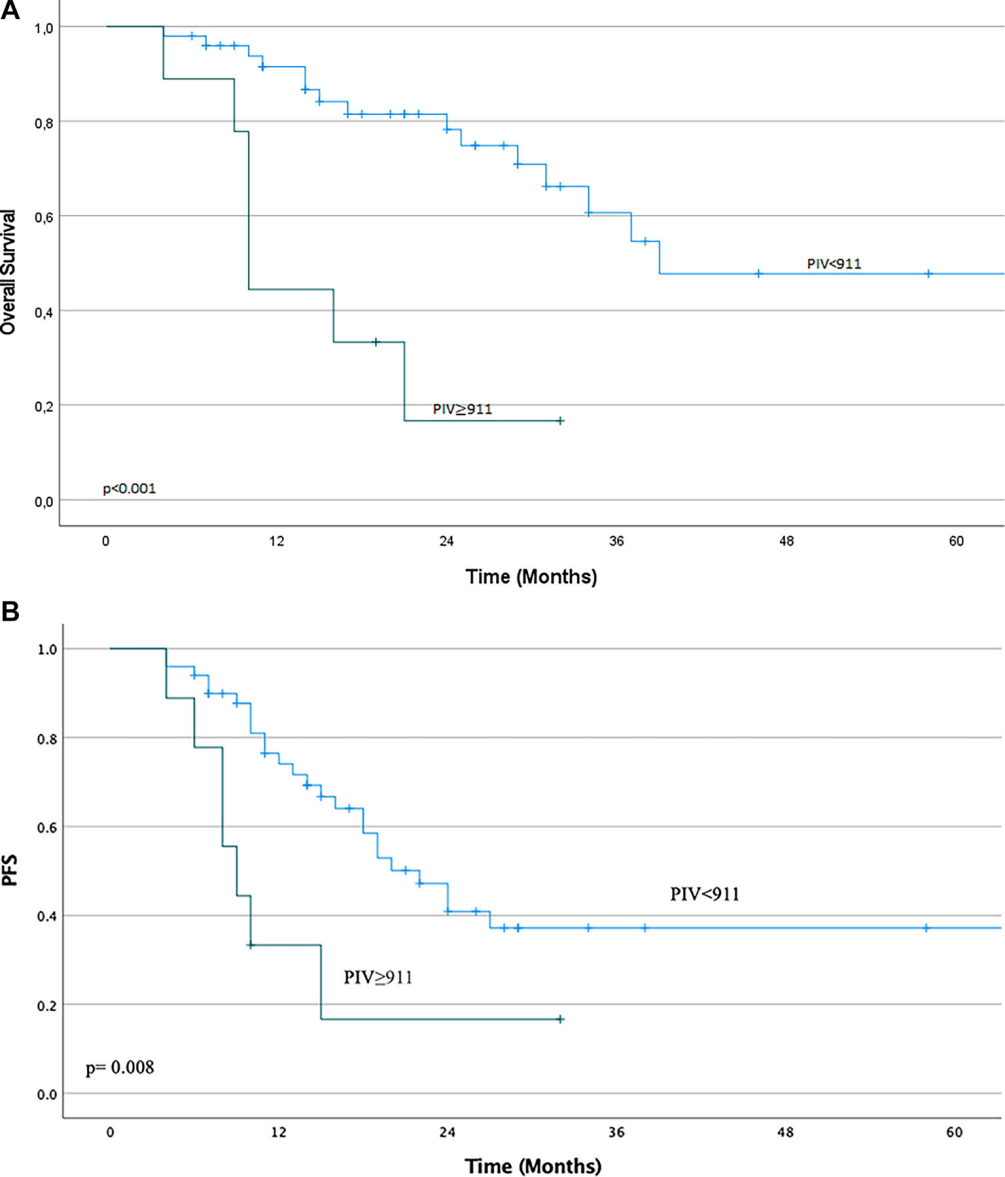
**The relationship between survival outcomes and PIV groups.** Kaplan–Meier survival curves comparing (A) OS and (B) PFS between patients with low (PIV < 911) and high (PIV ≥ 911) PIV. Patients with high PIV had significantly shorter OS (median 10 months vs 39 months, *P* < 0.001) and PFS (median 9 months vs 22 months, *P* ═ 0.008) compared to those with low PIV. PFS: Progression-free survival; PIV: Pan-immune inflammation value.

Overall progression-free survival (PFS) was significantly poorer in the high PIV group than in the low PIV group (median PFS: 9 months vs 22 months, *P* ═ 0.008). Similarly, the median OS time was 39 months for the low PIV group compared to 10 months for the high PIV group (*P* < 0.001). The relationships between survival outcomes and PIV groups are shown in Figure 2.

In the multivariate analysis, high pre-treatment PIV was significantly associated with poor ICPFS (*P* < 0.001, HR: 25.5, 95% CI: 4.21–154.7), PFS (*P* ═ 0.03, HR: 2.51, 95% CI: 1.06–5.95), and OS (*P* < 0.001, HR: 9.68, 95% CI: 2.64–35.4). Additionally, the timing of CRT impacted ICPFS and OS outcomes. When CRT was administered concurrently rather than sequentially, it was significantly associated with improved ICPFS (*P* ═ 0.02, HR: 8.26, 95% CI: 1.30–52.4) and OS (*P* ═ 0.004, HR: 6.65, 95% CI: 1.85–23.9). Although comorbidity was significantly associated with PFS in univariate analysis and showed borderline significance in the multivariate model (*P* ═ 0.051), it was not significantly associated with OS in univariate analysis (*P* ═ 0.056).

Although ECOG performance status differed significantly between the PIV groups, it was not found to be an independent prognostic factor for survival outcomes in multivariate analysis. Univariate and multivariate analyses for ICPFS are summarized in Table 2, and the analyses for PFS and OS are summarized in Table 3.

**Table 2:** Univariate and multivariate analyses of ICPFS

	**ICPFS**		
**Factors**	**Univariate**	**Multivariate**	**HR**
Gender (Male vs Female)	0.22	–	–
Age (<59 vs ≥59 years)	0.44	–	–
ECOG (0–1 vs 2)	0.72	–	–
Comorbidity (No vs Yes)	0.55	–	–
Smoking (No vs Yes)	0.45	–	–
Cht Regimen (Cis-Eto vs Carbo-Eto)	0.94		
Cht Cycles (<6 vs ≥6)	0.11		
CRT (Concurrent vs Sequential)	**0.01**	**0.02**	**8.26**
PIV (Low vs High)	**<0.001**	**<0.001**	**25.5**

ECOG: Eastern Cooperative Oncology Group; Cht: Chemotherapy; CRT: Chemoradiotherapy; PIV: Pan-immune inflammation value; HR: Hazard ratio; ICPFS: Intracranial progression-free survival.

**Table 3:** Univariate and multivariate analyses of OS and PFS

	**OS**			**PFS**		
**Factors**	**Univariate**	**Multivariate**	**HR**	**Univariate**	**Multivariate**	**HR**
Gender (Male vs Female)	0.17	–	–	0.27	–	–
Age (<59 vs ≥59 years)	0.74	–	–	0.60	–	–
ECOG (0–1 vs 2)	0.34	–	–	0.28	–	–
Comorbidity (No vs Yes)	0.05	–	–	**0.02**	**0.051**	**0.98**
Smoking (No vs Yes)	0.72	–	–	0.10	–	–
Cht Regimen (Cis-Eto vs Carbo-Eto)	0.09	–	–	0.23	–	–
Cht Cycles (<6 vs ≥6)	0.93	–	–	0.40	–	–
CRT (Concurrent vs Sequential)	**0.001**	**0.004**	**6.65**	0.17	–	–
PIV (Low vs High)	**<0.001**	**<0.001**	**9.68**	**0.013**	**0.036**	**2.51**

ECOG: Eastern Cooperative Oncology Group; Cht: Chemotherapy; CRT: Chemoradiotherapy; PIV: Pan-immune inflammation value; HR: Hazard ratio; OS: Overall survival; PFS: Progression-free survival.

## Discussion

This study aimed to evaluate the prognostic and predictive significance of PIV in patients with SCLC who underwent TRT followed by PCI. Through ROC curve analysis, the cut-off value for PIV was determined to be 911. Patients with a PIV < 911 demonstrated significantly higher rates of CR, ICPFS, PFS, and OS. These differences remained statistically significant even after multivariate analysis of OS, PFS, and ICPFS. These findings suggest that PIV may serve not only as a prognostic but also as a predictive marker.

Tumor progression and treatment responses are influenced by various immune cells, including neutrophils, platelets, monocytes, and lymphocytes. Neutrophils contribute to tumor progression by inhibiting the adaptive immune response through the release of high levels of reactive oxygen species and nitric oxide within the tumor microenvironment [20]. Additionally, neutrophils promote tumor angiogenesis, a process in which platelets and monocytes also play crucial roles [21–23]. In contrast, lymphocytes are vital for mounting an immune response against tumors [24]. Several biomarkers using these immune cells, such as the neutrophil-to-lymphocyte ratio (NLR), platelet-to-lymphocyte ratio (PLR), and systemic immune-inflammation index (SII), have been investigated for their prognostic impact [25–27]. For instance, in SCLC patients, a high NLR has been significantly associated with decreased OS and PFS [26]. Similarly, a high SII, which includes platelets, neutrophils, and lymphocytes, has been linked to worse OS outcomes in SCLC patients [28].

PIV was developed to assess the prognostic value of these four cell types in cancer patients [12]. The original study by Fuca et al. demonstrated that higher PIV levels were associated with poorer PFS (9.5 months vs 12.9 months; *P* < 0.001) and OS (21.6 months vs 34.4 months; *P* < 0.001) compared to lower PIV levels [12]. Although several studies have investigated the prognostic impact of PIV across various cancer types [14, 19, 29], only one study has focused specifically on SCLC patients who underwent TRT and PCI [4]. Our study is one of the few to examine the relationship between PIV and both OS and ICPFS in SCLC patients treated with TRT and PCI.

Our findings are consistent with a recent study by Kucuk et al. [4], which also investigated LS-SCLC. They found that lower pre-treatment PIV was associated with improved OS (25 months vs 14 months; *P* < 0.001). Additionally, Kucuk et al. reported significantly higher PFS rates in the low PIV group compared to the high PIV group, which aligns with our results. However, it is important to note that their study did not examine LRFS or ICPFS separately. They defined PFS as the duration between the start of treatment and either local progression or distant metastasis. In contrast, our study evaluated LRFS and ICPFS separately, and although we did not find a statistically significant difference in LRFS between the low and high PIV groups, we observed a statistically significant improvement in ICPFS for the low PIV group.

In addition to existing literature, we found a strong association between PIV and ICPFS. Recent studies have shown that neutrophils can disrupt the blood–brain barrier by secreting reactive oxygen species and proteases in the tumor microenvironment, while platelets facilitate brain metastasis by promoting extravasation [30]. As neutrophils and platelets are key components of the PIV formula, high PIV is therefore associated with intracranial progression.

Numerous studies evaluating the prognostic value of PIV have shown that lower PIV levels are associated with improved outcomes, findings consistent with our results [29, 31]. These findings demonstrate that the newly introduced marker, PIV, which incorporates four principal immune cells, can serve as a prognostic marker across various cancer types, regardless of histology, disease stage, or treatment modality.

While baseline comorbidities were modestly associated with PFS in our analysis, their impact did not extend to OS. Since comorbidity did not meet the inclusion threshold for the multivariate OS model and may be collinear with other clinical variables such as ECOG performance status, its influence is likely limited or indirect. Notably, the prognostic significance of PIV remained consistent, regardless of comorbidity. However, future prospective studies with larger cohorts and systematic assessment of comorbidities are needed to further clarify these relationships.

Previously published studies have reported varying PIV cut-off values. The threshold observed in our study appears relatively higher, which may be attributed to higher levels of systemic inflammation in lung cancer patients. Additional sensitivity analyses were performed using alternative PIV cut-off values. However, these analyses did not yield any statistically significant associations with survival outcomes, in contrast to the ROC-derived threshold of 911. These results are summarized in Table S1. In our ROC curve analysis, the AUC was found to be 0.60. Although this AUC indicates moderate discrimination, the high specificity (94%) suggests that PIV could be more useful in identifying low-risk patients rather than high-risk cases. Therefore, it may help identify patients who could potentially omit PCI.

This study is one of the few to evaluate the prognostic and predictive value of PIV in LS-SCLC and is the first to show an association between PIV and intracranial progression. However, there are several limitations. This trial has a retrospective design and includes a small sample size. The high PIV group, in particular, is small, with only nine patients included, which may reduce the statistical power of the findings and limit the ability to generalize the results. Nevertheless, we included all eligible patients who met the inclusion criteria to minimize selection bias. Additionally, the diversity of salvage therapies used after local or distant progression in SCLC may affect outcomes in both PIV groups. Given these limitations, multi-center prospective trials are necessary to better assess the prognostic value of PIV across various cancer types.

## Conclusion

The results of this study suggest that low PIV levels can be a favorable prognostic marker in LS-SCLC patients. It was found to be associated with improved OS and ICPFS. It can be used to determine prognosis and patient selection for PCI after the findings are supported by prospective trials.

## Supplemental data

**Table S1:** Sensitivity analyses of the association between PIV and survival outcomes using alternative cut-off values

**PIV Cut-off**	**OS**	**PFS**	**ICPFS**
624 (Median)	0.4	0.57	0.11
750	0.24	0.25	0.15
911 (ROC)	<0.001	0.008	<0.001

ROC: Receiver operating characteristic; OS: Overall survival; PFS: Progression-free survival; PIV: Pan-immune inflammation value; ICPFS: Intracranial progression-free survival.

## Data Availability

Research data are stored in an institutional repository and will be shared upon request to the corresponding author.
